# 0.79 ppm scale-factor nonlinearity whole-angle microshell gyroscope realized by real-time calibration of capacitive displacement detection

**DOI:** 10.1038/s41378-021-00306-6

**Published:** 2021-10-13

**Authors:** Jiangkun Sun, Sheng Yu, Yongmeng Zhang, Qingsong Li, Xiang Xi, Kun Lu, Xuezhong Wu, Dingbang Xiao

**Affiliations:** National University of Defense Technology, Changsha, 410073 China

**Keywords:** Electrical and electronic engineering, Sensors

## Abstract

Whole-angle gyroscopes have broad prospects for development with inherent advantages of excellent scale factor, wide bandwidth and measurement range, which are restrictions on rate gyroscopes. Previous studies on the whole-angle mode are based mostly on the linear model of Lynch, and the essential nonlinearity of capacitive displacement detection is always neglected, which has significant negative effects on the performance. In this paper, a novel real-time calibration method of capacitive displacement detection is proposed to eliminate these nonlinear effects. This novel method innovatively takes advantage of the relationship between the first and third harmonic components of detective signals for calibration. Based on this method, the real-time calibration of capacitive displacement detection is achieved and solves the problems of traditional methods, which are usually related to the vibration amplitude, environmental variations and other factors. Furthermore, this novel calibration method is embedded into a whole-angle control system to restore the linear capacitive response in real time and then combined with a microshell resonator for the first time to exploit the enormous potential of an ultrahigh *Q* factor and symmetric structure. The effectiveness is proven because the angle drift is reduced significantly to improve the scale-factor nonlinearity by 14 times to 0.79 ppm with 0.0673°/h bias instability and a 0.001°/s rate threshold, which is the best reported performance of the MEMS whole-angle gyroscope thus far. More importantly, this novel calibration method can be applied for all kinds of resonators with the requirement of a linear capacitive response even under a large resonant amplitude.

## Introduction

High-performance micromachined vibratory gyroscopes based on Coriolis force coupling have received increased attention in the applications of high-end industrial, aerospace, robotics and unmanned systems. Conventional high-performance micromachined gyroscopes usually adopt rate mode to measure the angular rate by driving one mode to vibrate and detect the Coriolis-induced vibration of the second mode^1–4^. Currently, the restrictions of the rate mode are becoming increasingly obvious since the performance of rate measurements is severely affected by nonlinearity in the scale factor and limited measurement bandwidth^5–7^. In contrast, in the whole-angle mode, the mechanical resonator acts as a “mechanical integrator” of angular rate, producing the output angle that follows the rotation directly. To achieve this behavior, the vibration mode must be allowed to process freely in response to the Coriolis force. Then, the pattern angle of the vibration mode can be calculated as the output angle that tracks the input rotation in real time. The inherent advantages of the whole-angle mode are critically essential for the application of gyros. First, the scale factor of the whole-angle mode is related only to the structure of the resonator and insensitive to environmental variations, resulting in the highly stable characteristic of the scale factor. In addition, the gyro under whole-angle mode theoretically has unlimited bandwidth and measurement range, which may be compromised by the bandwidth of the electronic control loop in practice^8–10^. Despite these attractive characteristics of the whole-angle mode, its performance relies heavily on the stiffness and damping symmetry and may not be suitable for a great deal of MEMS resonators^11,12^. A microshell resonator with many outstanding characteristics is an excellent candidate for the whole-angle mode^4,13–16^. Specifically, a microshell resonator fabricated from fused silica with low thermoelastic damping has the potential to achieve an ultrahigh *Q* factor of over 5 million and a ring-down time of 295 s, as reported in the reference^4^. Furthermore, the inherent symmetrical structure of the microshell resonator has excellent symmetrical characteristics, and the stiffness asymmetry can be further reduced by mechanical trimming to obtain a perfect resonator^17,18^. Above all, combining a microshell resonator and whole-angle mode is a perfect collaboration to realize high-performance micromachined gyroscopes.

The major limitation for the performance of the whole-angle mode is the angle drift, which has major effects on the angle-measurement accuracy and scale-factor nonlinearity^19–21^. Normally, the control algorithms and error model of the whole-angle mode are based on the linear dynamic model proposed by Lynch, which ignores some nonlinear features of gyroscopes, such as the geometric nonlinear effect, capacitive electrostatic actuation and detection nonlinearity^22–24^. This linear model reveals the existence of a 2*θ* angle-dependent harmonic component in the angle drift resulting from stiffness and damping asymmetry, which can be compensated by the quadrature nulling loop and velocity feedback control, respectively^8,19,25^. However, related works have demonstrated the existence of a 4*θ* angle-dependent harmonic in the angle drift derived from nonlinear dynamics, and it cannot be compensated simply via feedback control, which will have great negative effects on the performance^20,26^. Some nonlinear compensation and correction methods have been proposed to eliminate nonlinear effects^27,28^. However, the previous methods need to search for a suitable parameter offline for calibration, which is time-consuming and arduous. It is even more disappointing that it cannot adapt to different control parameters and environmental variations.

In this paper, a novel calibration method for capacitive displacement detection nonlinearity is proposed based on a harmonic-component relationship to restore the linear response in real time. This novel method is first applied to simple closed-loop control to verify its effectiveness. Then, the novel method is embedded into the whole-angle control system to eliminate the nonlinear effects of capacitive displacement detection. With this novel method, the 4*θ* angle-dependent harmonic component in the drift rate has been removed, and the scale-factor nonlinearity of the microshell resonator gyroscope has been improved to 0.79 ppm. Furthermore, this novel method can also be applied to all kinds of capacitive resonators to increase the amplitude with a linear response.

## Results

### Device architecture and nonlinearity of capacitive displacement detection

**Fig. 1:**
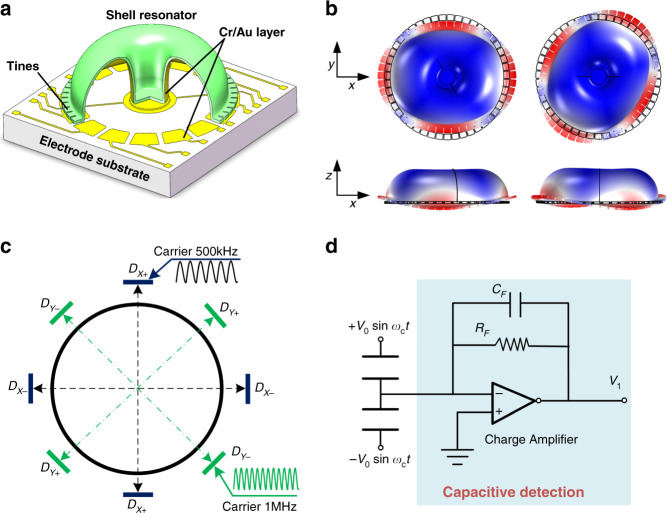
Device architecture and capacitive displacement detection. **a** Structural view of the microshell resonator. **b** Wineglass modes. **c** Electrode configuration of whole-angle mode. **d** Schematic diagram of differential capacitive displacement detection.

The microshell resonator, shown in Fig. 1a, is bonded to the planar electrode substrate through the anchor. Both the resonator and electrode substrates are made of fused silica. This architecture of fully fused silica provides stable resonance properties within the whole temperature range, which significantly reduces errors caused by thermal expansion. The height and diameter of this resonator are ~4 mm and 12 mm, respectively. As illustrated in Fig. 1b, the *n* = 2 wineglass modes are selected as working modes for the whole-angle mode. Due to the presence of in-plane deformation and out-of-plane deformation in the vibration of wineglass modes simultaneously, out-of-plane electrodes are used to drive or sense the vibration of the wineglass modes. There are 48 rectangular shape tines around the perimeter of the resonator to collaborate with 16 separated electrodes for capacitive transduction. The capacitive gap *d*_*0*_ between the planar electrodes and the perimeter of the resonator is ~20 μm. For the whole-angle mode, the gyroscope will need two sets of separated electrodes for actuation and detection of mode *X* and mode *Y*. To satisfy this requirement with the limited electrodes of the microshell resonator, the driving signals of mode *X* and mode *Y* mixed with the high-frequency carrier are applied on electrodes *D*_*X*_ and *D*_*Y*_. In addition, the sensing signals of the two modes are obtained from the microshell resonator and are distinguished by the modulation of two high-frequency carriers, as shown in Fig. 1c. Differential actuation and detection are used in this whole-angle mode, and schematic diagrams are shown in Fig. 1d to analyze the nonlinear behavior of capacitive responses.

The vibration signals are detected by differential capacitive detection with carrier demodulation, and the carrier frequency *ω*_*c*_ is much larger than the resonant frequency of the gyroscope. Hence, the variation in differential capacitance after Fourier expansion can be expressed as

1 $${\Delta}C = \frac{{\varepsilon A}}{{d_0 - x}} - \frac{{\varepsilon A}}{{d_0 + x}} \approx \frac{{2\varepsilon A}}{{d_0^2}}\left[ {1 + \left( {\frac{x}{{d_0}}} \right)^2} \right]x$$

where *x* represents the vibration displacement of the resonator. Therefore, the detected signal after carrier demodulation can be calculated as

2 $$V_1 = - \frac{{V_0}}{{C_{FB}}}{\Delta}C = k_s\left[ {1 + \left( {\frac{x}{{d_0}}} \right)^2} \right]x$$

where $k_s = - 2\varepsilon AV_0/C_{FB}d_0^2$ can be regarded as the detective gain of the capacitance. In addition, *V*_0_ is the amplitude of the carrier, and *C*_*FB*_ represents the feedback capacitance. Equation (2) shows that the detective signal contains a nonlinear component, which represents the nonlinear effects of capacitive displacement detection and affects the detection accuracy.

Taking nonlinear capacitive displacement detection into consideration a certain deviation generates in the real detection signal of the resonator. If the displacement of vibration is expressed as *x* = *x*_0_cos(*ωt* + *φ*) and substituted into Eq. (2), the corresponding output signal can be written as

3 $$\begin{array}{lll}V_1& =& k_s\left[ {1 + \frac{3}{4}\left( {\frac{{x_0}}{{d_0}}} \right)^2} \right]x_0\cos \left( {\omega t + \varphi } \right) + k_s\frac{{x_0^3}}{{4d_0^2}}\cos 3\left( {\omega t + \varphi } \right)\\& = &\left[k_{s} x_{0}{+3}{\Pi}\right]\cos \left( {\omega t + \varphi } \right) + {\Pi}\cos 3\left( {\omega t + \varphi } \right)\end{array}$$

where ${\Pi} = k_sx_0^3/4d_0^2$. The harmonic component of 3*ω* can be eliminated after demodulation, and the effective signal entering the control system is given by

4 $$V_e = {{{\mathrm{[}}}}k_sx_0{{{\mathrm{ + 3}}}}{\Pi}{{{\mathrm{]}}}}\cos \left( {\omega t + \varphi } \right)$$

Equation (4) also shows that the displacement of vibration becomes overestimated compared with the linear response, and 3Π is the component that needs to be removed.

### Real-time calibration of capacitive detection nonlinearity

The basic idea of this novel calibration method of capacitive displacement detection is based on the relationship between the first and third harmonic components of the detection signal. According to Eq. (4), the nonlinear element 3Π must be removed to calibrate the nonlinearity of capacitive displacement detection. Equation (3) also shows that the extra amplitude 3Π of the first harmonic component is exactly three times as large as the amplitude Π of the third harmonic component, indicating that the superfluous amplitude 3Π in the first harmonic component can be identified in real time as long as the amplitude Π in the third harmonic component can be obtained. Therefore, the nonlinear element of the first harmonic component 3Π can be eliminated in real time based on the analysis above.

**Fig. 2:**
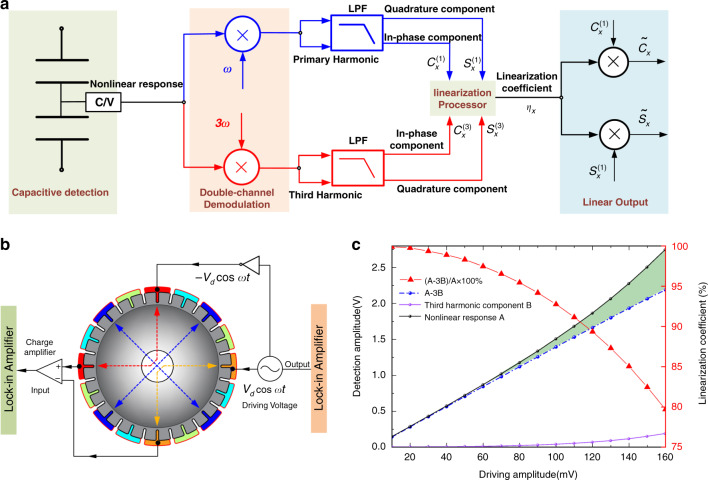
Illustration of real-time calibration method for capacitive detection nonlinearity. **a** Schematic diagram of this novel real-time calibration method. **b** Open-loop platform to study the nonlinear behavior of capacitive detection. **c** Experimental results to verify this novel calibration method.

The detailed implementation procedures of the signal processor to realize nonlinear elimination are illustrated in Fig. 2a. Double-channel demodulation is adopted to synchronously obtain the first and third harmonic components. After low-pass filtering, the in-phase and quadrature components of the first and third harmonic components$c_x^{(1)},s_x^{(1)},c_x^{(3)}s_x^{(3)}$ are obtained to calculate the linearization coefficient *η*_*x*,_ which can be defined as

5 $$\eta _x = \frac{{A - 3B}}{A} = \frac{{\sqrt {\left[ {c_x^{(1)}} \right]^2{{{\mathrm{ + }}}}\left[ {s_x^{(1)}} \right]^2} - 3\sqrt {\left[ {c_x^{(3)}} \right]^2 + \left[ {s_x^{(3)}} \right]^2} }}{{\sqrt {\left[ {c_x^{(1)}} \right]^2 + \left[ {s_x^{(1)}} \right]^2} }} \le 1$$

where *A* and *B* are the amplitudes of the first and third harmonic components, respectively. *A-3B* represents the amplitude of the linear response obtained by eliminating the amplitude 3Π. Therefore, this linearization coefficient is the ratio between the linear response and original nonlinear response, and its value is less than or equal to one. To obtain the linear response, we need only this linearization coefficient to calibrate the original nonlinear response. Therefore, the linearization output of the in-phase and quadrature components are calculated as $\tilde c_x = \eta _xc_x^{(1)}$ and $\tilde s_x = \eta _xs_x^{(1)}$ in real time.

To verify this novel method, a simple experimental platform of open-loop control is set up to study the nonlinear behavior of capacitive detection, as shown in Fig. 2b. Differential actuation and detection are applied on the electrodes of the microshell resonator through a lock-in amplifier (LIA, Zurich Instruments HF2LI). In addition, the driving amplitude is proportional to the vibration amplitude due to the phase-locked loop (PLL). Therefore, different driving amplitudes are applied to obtain the corresponding vibration amplitudes. For every driving amplitude, the first and third harmonics of the PLL are employed to demodulate the detection signal at the same time. Then, the amplitudes of the first and third harmonic components can be obtained. As illustrated in Fig. 2c, the nonlinear response A obeys the linear rule when the driving amplitude is very small. However, the nonlinear response A has an obvious upward trend with increasing driving amplitude due to the nonlinear offset, as explained in Eq. (4). The amplitude of third harmonic component B is also observed to increase at the same time. Furthermore, a nonlinear calibration is also carried out to restore the linear response by calculating A-3B. The response after nonlinear calibration shows an excellent linear trend, and its goodness of fit *R*^2^ = 0.9999. Nonlinear calibration has been proven to be effective, and the linear response can be restored by removing the nonlinear offset. The linearization coefficient η_x_, also calculated according to Eq. (5), decreases gradually with increasing driving amplitude and ranges from 1 to 0.8, representing the nonlinear degree of capacitive displacement detection. In conclusion, the open-loop experimental results above have proven the effectiveness of this novel nonlinear calibration method, which has provided a solid foundation for its application under the whole-angle mode in the next chapter.

### Effects of capacitive detection nonlinearity under whole-angle mode

**Fig. 3:**
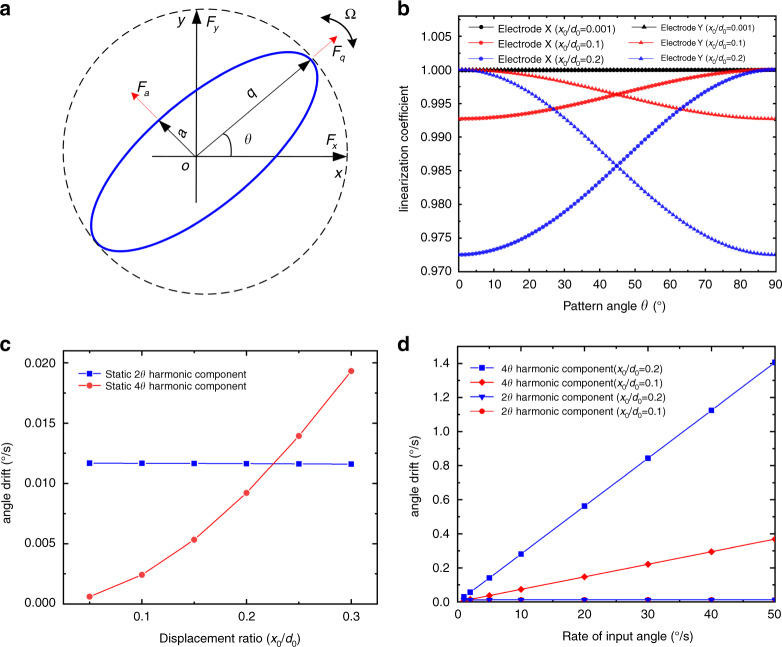
Effects of capacitive detection nonlinearity. **a** General elliptic orbit and procession of the vibration pattern. **b** Simulation results of the linearization coefficient under different pattern angles. **c** Simulation results of the angle drift rate under different rotation rates. **d** Experimental results of the angle drift rate under different rotation rates.

For the microshell resonator in this study, the mechanical nonlinearity can be neglected because the vibration amplitude is very small compared with the dimension of this resonator. Hence, electrostatic nonlinearity is the major factor that may influence the performance of the gyroscope. In rate mode, the vibration mode works in a fixed position with a constant amplitude. Hence, capacitive detection nonlinearity is maintained at the same level all the time, which will not have great effects on the performance. In the whole-angle mode, the vibration pattern in Fig. 3a can be regarded as the composition of mode X and mode Y, as shown in Fig. 1b. During rotation, the amplitudes of the two modes modify simultaneously when the pattern angle *θ* processes under the whole-angle mode. The nonlinearity of capacitive detection will also change at the same time. This special capacitive nonlinearity characteristic under whole-angle mode will be explained in detail as follows.

To achieve the behavior of the whole-angle mode, external forces are applied to maintain the length of the semimajor axis *a* and suppress the semiminor axis *q* to zero, as shown in Fig. 3a. Thus, the pattern angle *θ* of the vibration mode can process freely responding to the input rotation. The sensed displacements in the *x-* and *y*-axes can be decomposed into in-phase and in-quadrature components with *ωt* + *φ* as follows:

6 $$\begin{array}{l}x = a\cos \theta \cos (\omega t + \varphi ) - q\sin \theta \sin (\omega t + \varphi )\\ y = a\sin \theta \cos (\omega t + \varphi ) + q\cos \theta \sin (\omega t + \varphi )\end{array}$$

Equation (6) shows that the sensed displacements in the *x-* and *y*-axes modify at the same time as the pattern angle *θ* proceeds. Hence, the nonlinearity of electrodes in the *x-* and *y*-axes also alters along with the modification of sensed displacements. This is a unique feature different from other control systems and will result in major effects on the performance. As shown in Fig. 3b, the variation in linearization coefficients is calculated by numerical analysis. Because the gap *d*_*0*_ is a constant, different vibration amplitudes *x*_*0*_ can represent different degrees of capacitive detection nonlinearity. When the vibration amplitude is small (*x*_*0*_/*d*_*0*_ = 0.001), the linearization coefficients of electrodes *X* and *Y* always remain at ~1 under different pattern angles, representing a linear response at all times. However, the linearization coefficients of electrodes *X* and *Y* have the opposite variation trend under large vibration amplitudes (*x*_*0*_/*d*_*0*_ = 0.1), representing large nonlinearity when the pattern angle *θ* changes from 0° to 90°. Specifically, the nonlinearity of electrode *X* is the largest, but electrode *Y* is in a linear response status when the pattern angle *θ* = 0°. In contrast, the nonlinearity of electrode *Y* is in the strongest nonlinear status, while the response of electrode *X* is still linear when the pattern angle *θ* = 90°. In particular, the responses of electrodes *X* and *Y* have the same nonlinearity when the pattern angle *θ* = 45°. The minimum linearization coefficient decreases from 0.9925 to 0.9725 with increasing vibration amplitude from *x*_*0*_/*d*_*0*_ = 0.1 to *x*_*0*_/*d*_*0*_ = 0.2. Above all, the nonlinearity of electrodes *X* and *Y* will be modified in a reverse way at the same time under the precession of pattern angle *θ*, which will have major influences on the performance.

The nonlinearity of capacitive detection results mainly in angle drift on the performance, which has been derived in detail in the Supplementary Material. As illustrated in ref. ^27^, the nonlinear effects of capacitive electrostatic actuation on the angle-drift error can be removed by quadrature nulling and have been ignored in this paper. According to the Supplementary Material, the angle drift rate resulting from capacitive detection nonlinearity can be expressed as:

7 $$\begin{array}{l}\dot \theta _{drift} = \dot \theta _{damp\_asy} + \dot \theta _{nonlinear}\\ \dot \theta _{damp\_asy} = \frac{1}{2}{\Delta}\left( {\frac{1}{\tau }} \right)\sin 2\left( {\theta - \theta _\tau } \right)\\ \dot \theta _{nonlinear} = \frac{1}{\tau }\tan \delta \theta - \kappa {\Omega}\frac{{d\left( {\delta \theta } \right)}}{{d\theta }} - \kappa {\Omega}\frac{1}{{\Lambda}}\frac{{\partial {\Lambda}}}{{\partial \theta }}\tan \delta \theta \end{array}$$

where 1/*τ* and Δ(1/*τ*) represent the mean damping and damping asymmetry, respectively. In addition, $\delta \theta$is the angle-estimated error of pattern angle *θ* resulting from capacitive detection nonlinearity. ${\Lambda}$calculated from the equation in the Supplementary Material is the solution of the semimajor axis *a* and is related to the pattern angle *θ*.

Equation (7) shows that the angle drift rate consists of two parts. The former induced by damping asymmetry will generate only a 2*θ* harmonic component. However, the latter, coming from capacitive detection nonlinearity, will produce a 4*θ* harmonic component due to the existence of$\delta \theta$, which can be observed clearly from the numerical simulation in the Supplementary Material. Equation (7) also shows that the angle drift rate $\dot \theta _{nonlinear}$ representing the 4*θ* harmonic component is proportional to the input rate Ω.

To investigate the effects of capacitive detection nonlinearity on gyro performance, numerical simulations are carried out. Owing to the angle-dependent characteristic of the angle drift rate, one can fit the Fourier series ($f(\theta ) = 0.5a_0 + \mathop {\sum}\nolimits_{n = 1}^\infty {a_n\cos (2n\theta )} + \mathop {\sum}\nolimits_{n = 1}^\infty {b_n\sin (2n\theta )}$) to each simulation result to determine the amplitudes of the 2*θ* and 4*θ* harmonic components. As shown in Fig. 3c, the 2*θ* harmonic component of the angle drift rate remains the same and independent of the rotation rates. However, the 4*θ* harmonic component coming from capacitive detection nonlinearity is proportional to the rotation rate. In addition, the growth rate of the 4*θ* harmonic component increases when *x*_*0*_/*d*_*0*_ changes from 0.035 to 0.1. The angle drift rate coming from the 4*θ* harmonic component has already far outweighed the angle drift rate induced by the 2*θ* harmonic component when the rotation rate is relatively large. Hence, the removal of the 4*θ* harmonic component is of great importance to improve the performance of the gyro. At the same time, experiments are also carried out to verify the numerical simulation results, as illustrated in Fig. 3d, under different vibration amplitudes *x*_*0*_ = 448 mV and *x*_*0*_ = 1228 mV. The simulation and experimental results are consistent with each other. In summary, the 4*θ* harmonic component in the angle drift rate comes from capacitive detection nonlinearity and is proportional to the input rate Ω. Furthermore, it cannot be simply compensated by a feedback control loop. Therefore, the calibration of capacitive displacement detection is the only way to remove the effects of the 4*θ* harmonic component.

### Implementation of real-time calibration under whole-angle mode

**Fig. 4:**
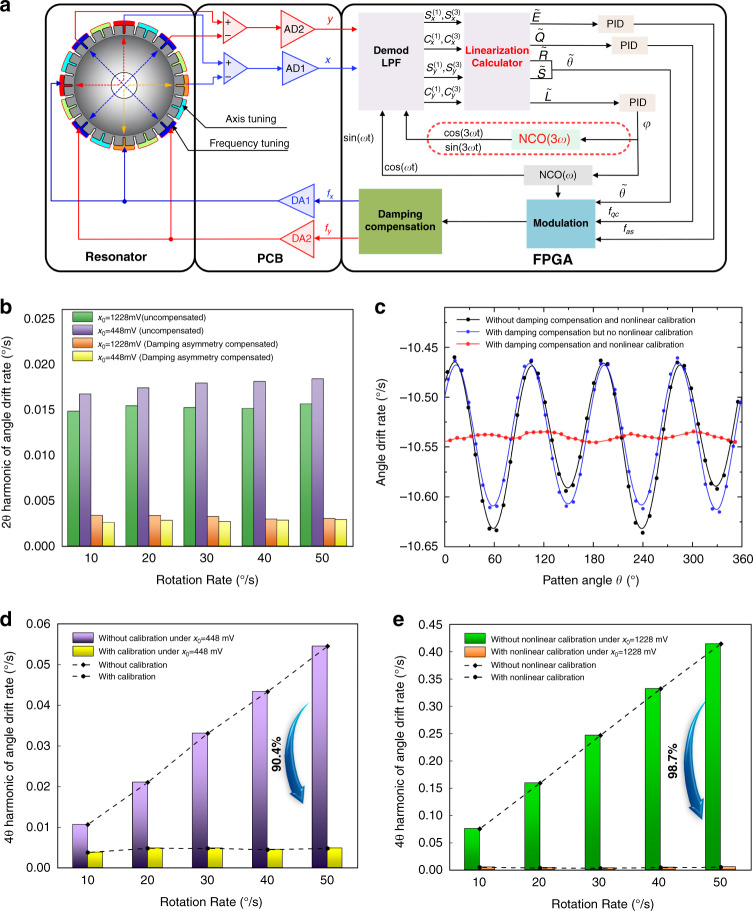
Implementation of real-time calibration under whole-angle mode. **a** Schematic illustration of the complete whole-angle mode with the linearization process. **b** 2*θ* harmonic component of the drift rate before and after damping asymmetry compensation without nonlinear calibration. **c** Angle drift rate under rotation rate of 10°/s under *x*_0_ = 1228 mV. **d**, **e** 4*θ* harmonic component of drift rate before and after nonlinear calibration under *x*_0_ = 448 mV and *x*_0_ = 1228 mV, respectively.

Based on the novel nonlinear calibration method in this paper, a complete control system of the whole-angle mode was designed, as illustrated in Fig. 4a. Apart from a microshell resonator, this platform also consists of two printed circuit boards (PCBs). One PCB contains analog amplifiers for the detection and actuation of the resonator’s vibration and mixed-signal electronics: digital-to-analog converters (DACs) and analog-to-digital converters (ADCs). The residual part is the FPGA platform for digital signal processing and implementation of the controllers.

8 $$\begin{array}{l}\tilde E = \tilde c_x^2 + \tilde s_x^2 + \tilde c_y^2 + \tilde s_y^2 = a^2 + q^2\\ \tilde Q = 2\left( {\tilde c_x\tilde s_y - \tilde c_y\tilde s_x} \right) = 2aq\\ \tilde L = 2\left( {\tilde c_x\tilde s_x + \tilde c_y\tilde s_y} \right) = \left( {a^2 - q^2} \right)\sin 2\delta \phi \\ \tilde R = \tilde c_x^2 + \tilde s_x^2 - \tilde c_y^2 - \tilde s_y^2 = \left( {a^2 + q^2} \right)\cos 2\theta \\ \tilde S = 2\left( {\tilde c_x\tilde c_y + \tilde s_x\tilde s_y} \right) = \left( {a^2 - q^2} \right)\sin 2\theta \\ \tilde \theta = \frac{1}{2}\arctan \left( {\tilde S/\tilde R} \right)\end{array}$$

To realize the linearization of capacitive displacement detection, only some simple modifications on the FPGA platform are enough. First, an extra numerically controlled oscillator (NCO) for demodulation of the third harmonic component has been added into this system to obtain in-phase and quadrature components of detective electrodes *X* and *Y*, namely$c_x^{(3)},s_x^{(3)},c_y^{(3)},s_y^{(3)}$. Second, a linearization calculator according to Eq. (5) is applied to obtain the linearization coefficients *η*_*x*_ and *η*_*y*_ for detective electrodes *X* and *Y*, respectively. Finally, the linearization output of the in-phase and quadrature components are calculated as$\tilde c_x = \eta _xc_x^{(1)}$,$\tilde s_x = \eta _xs_x^{(1)}$,$\tilde c_y = \eta _yc_y^{(1)}$and $\tilde s_y = \eta _ys_y^{(1)}$. Then, the calibrated control variables $\tilde E,\tilde Q,\tilde L,\tilde R\& \tilde S$ for energy, quadrature, PLL control loops and pattern angle$\tilde \theta$can be calculated as Eq. (8).

Then, experiments are carried out to investigate the effects of capacitive detection nonlinearity under different amplitudes and rotation rates. In the data processing of these experiments, the angle output is calculated as 3.7*θ to make it close to the input rotation Ω and the angle drift rate is obtained by the differential operation of angle output. As shown in Fig. 4b, the 2θ harmonic component of the drift rate under a large vibration amplitude (x_0_ = 1228 mV) is almost the same as that of a small vibration amplitude (x_0_ = 448 mV) under different rotation rates. The small difference in the 2θ harmonic component results from the gain errors of detection and actuation under different vibration amplitudes^29^, indicating that the 2θ harmonic component of the drift rate has no relationship with the vibration amplitude and rotation rate. Furthermore, the 2θ harmonic component of the angle drift rate can be effectively eliminated by the compensation of damping asymmetry, and this compensation method is explained in the Supplementary Material. After compensation for the damping asymmetry, the 2θ harmonic component is reduced to less than 0.003°/s. However, damping asymmetry compensation has no effect on the 4θ harmonic component of the drift rate, and the removal of the 4θ harmonic component can only be realized by the calibration of capacitive detection nonlinearity. Therefore, the 4θ harmonic component dominates in the angle drift rate after damping asymmetry compensation. For example, the drift-rate curve under a rotation rate of 10°/s without damping asymmetry, and nonlinear calibration is composed of a 2θ harmonic component of 0.021°/s and a 4θ harmonic component of 0.076°/s, as shown in Fig. 4c. However, almost only the 4θ harmonic component of 0.076°/s is left in the drift-rate curve after damping asymmetry compensation. Furthermore, the 4θ harmonic component is reduced to ~0.004°/s after the calibration of capacitive detection nonlinearity, and the angle drift rates under different pattern angles are almost the same. The effectiveness of nonlinear calibration has also been verified under different rotation rates and vibration amplitudes. As illustrated in Fig. 4d, e, the linear growth trends under different rotation rates were suppressed with this nonlinear calibration method. Moreover, the 4θ harmonic components have decreased by 90.4% and 98.7%, respectively, and the remaining parts are only ~0.004°/s left when x_0_ = 448 mV and x_0_ = 1228 mV. Above all, the 4θ harmonic component induced by capacitive detection nonlinearity is no longer related to the vibration amplitude and rotation rate after nonlinear calibration. Therefore, the experimental results above prove that this nonlinear calibration method of capacitive detection nonlinearity is effective and that most 4θ harmonic components can be eliminated to improve the performance of the gyro.

### Gyroscope performance characterization

**Fig. 5:**
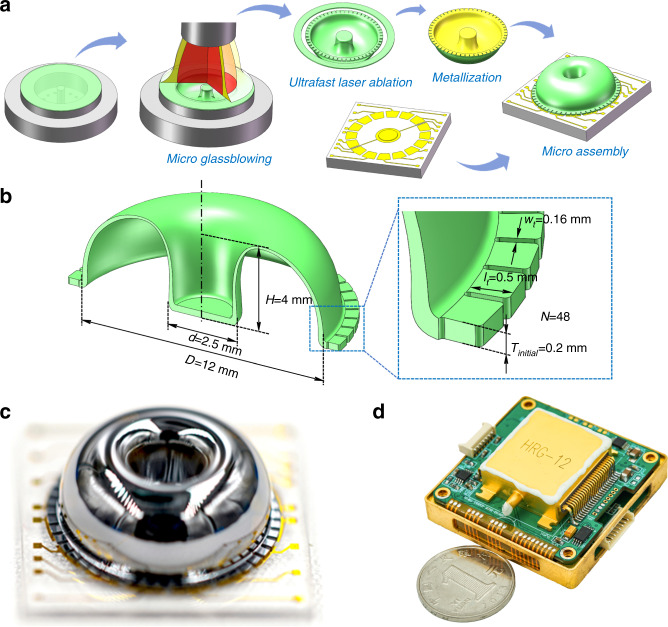
Illustration of the micro-shell resonator gyroscope. **a** Schematic illustration of the microshell resonator fabrication process. **b** Geometry parameters include the shell diameter D, height H, anchor diameter d, initial thickness T_initial_, tine length l_t_, tine gap w_t_ and tine number N. **c** Photograph of the accomplished resonator. **d** Photograph of a complete whole-angle microshell gyroscope.

The fabrication of a microshell resonator is of great importance for the performance of the gyroscope. Figure 5a provides a schematic illustration of the fabrication process for microshell resonators. The substrate is placed on a graphite mold and mounted by vacuum pressure. Then, the forming process starts by turning on the whirling platform and aligning the blowing torch toward the center of the graphite mold, and the propane-oxygen torch provides a high temperature above 1700 °C to heat the fused silica substrate. Then, ultrafast laser ablation is introduced to detach the resonator from the substrate due to its unique advantages in micromachining transparent materials. Finally, the resonator is bonded to the substrate through microassembly after metallization^15^. A resonator with teeth-like tines along the perimeter has the advantages of significantly improving the vibration mass and capacitance area of the resonator and simplifying mechanical trimming by minimizing the effect of mass removal on the stiffness^30^. The geometric parameters of the microshell resonator, including the shell diameter *D*, height *H*, anchor diameter *d*, initial thickness *T*_*initial*_, tine length *l*_*t*_, tine gap *w*_*t*_ and tine number *N*, are illustrated in Fig. 5b. The accomplished resonator is illustrated in Fig. 5c, and the metallic packaging method is adopted to maintain a stable vacuum environment by keeping the intracavity pressure at 0.01 Pa. A complete whole-angle microshell gyroscope including a packaged microshell resonator and electronic control system is shown in Fig. 5d.

**Fig. 6:**
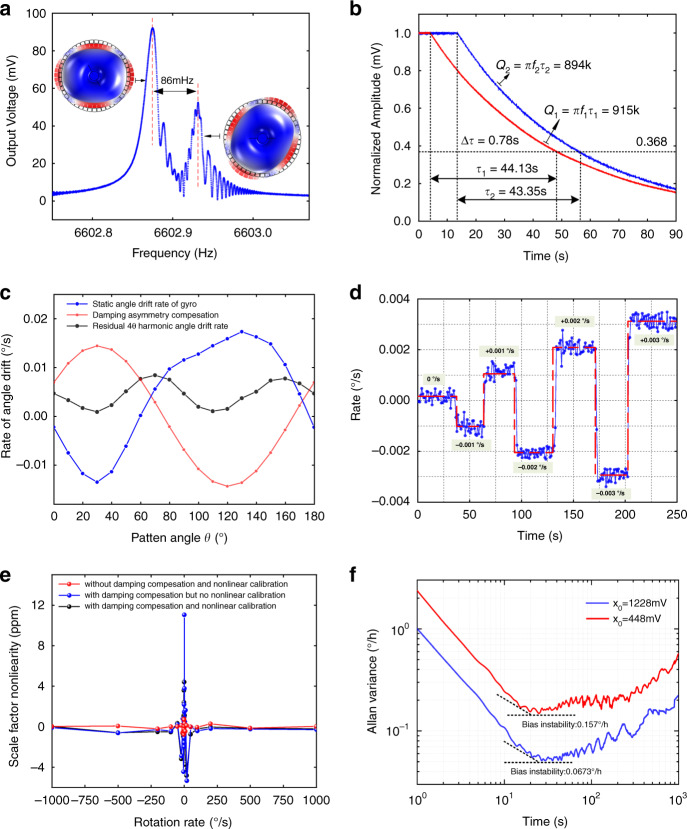
Performance characterization. **a**, **b** Frequency response and ring-down tests of the microshell resonator. **c** Angle drift rate under whole-angle mode without rotation. **d** Rate threshold test of the whole-angle microshell resonator gyroscope. **e** Scale-factor nonlinearity under damping asymmetry compensation and nonlinear calibration. **f** Allan deviations of different vibration amplitudes.

To characterize the performance of the microshell resonator gyroscope, frequency response and ring-down tests are carried out. As shown in Fig. 6a, b, the frequency mismatch between the two modes is ~86 mHz after mechanical trimming, and the ring-down times of the two modes are 44.13 s and 43.35 s, respectively, which demonstrates the excellent symmetry of this microshell resonator. In addition, the angle drift rate under the different pattern angles is measured to be less than ±0.02°/s, as illustrated in Fig. 6c, which can be used to evaluate the damping asymmetry of the microshell resonator. Then, damping asymmetry compensation is applied to remove the 2θ harmonic component from the angle drift rate, and the residual part is shown as a 4θ harmonic component of the drift rate. Figure 6d demonstrates the responses of the microshell gyroscope under rotations of ±0.001°/s, ±0.002°/s and ±0.003°/s, which proves that the rate threshold is below 0.001°/s. Furthermore, the nonlinearity of the scale factor is tested under ±0.1°/s, ±0.2°/s, ±0.5°/s, ±1°/s, ±2°/s, ±5°/s, ±10°/s, ±20°/s, ±50°/s, ±100°/s, ±200°/s, ±500°/s and ±1000°/s. For every rotation rate, the gyroscope acquired output data for 30 s with a sampling rate of 1 kHz. The performance of the microshell resonator gyroscope under whole-angle mode is limited under slow-speed rotation due to angle drift. However, this gyroscope is quite suitable for high-speed rotation because the periodical drift error can be averaged to zero. Hence, the maximum scale-factor nonlinearity is distributed in the slow-speed regions. As shown in Fig. 6e, the scale-factor nonlinearity is ~11.05 ppm without damping asymmetry compensation and nonlinear calibration. If the damping asymmetry is compensated, the 2θ harmonic component of the drift rate will be removed, and the scale-factor nonlinearity decreases to 4.86 ppm without linearization calibration. Especially when the 2θ and 4θ harmonic components of the drift rate are eliminated with damping asymmetry compensation and nonlinear calibration, the scale-factor nonlinearity will be just 0.79 ppm, which is an improvement of 14 times. Meanwhile, the bias instability increases from 0.157°/h to 0.0673°/h along with the increment of vibration amplitude, due mainly to the improvement of signal-to-noise ratio (SNR). Without this nonlinear calibration method, there is a compromise between SNR and excellent scale-factor nonlinearity. For example, the SNR will severely restrict the performance of the gyroscope because the resonator must work under a very small amplitude due to the micron-level gaps of the MEMS resonator if excellent scale-factor nonlinearity is required. Therefore, the advantages of better bias instability and excellent scale-factor nonlinearity can be achieved at the same time under a large vibration amplitude with this novel nonlinear calibration method of capacitive detection.

## Conclusions

This paper presents a novel method of nonlinear calibration based on the relationship between the first and third harmonic components of detective signals. A complete control loop under whole-angle mode is established to restore the capacitive linear response in real time to remove the effects of capacitive detection nonlinearity by eliminating the 4*θ* harmonic component of the angle drift rate. Meanwhile, experiments are also carried out to verify the effectiveness of this novel method. Combining this novel method with the whole-angle control system, the first practical whole-angle microshell resonator gyroscope is realized with excellent performance. The bias instability is improved from 0.157°/h to 0.0673°/h due to the enhancement of the SNR. Furthermore, the rate threshold is tested to be lower than 0.001°/s, and the scale-factor nonlinearity is improved ~14 times to 0.79 ppm with damping asymmetry and nonlinear calibration. This is the best reported performance for MEMS whole-angle gyroscopes thus far, and the collaboration of microshell resonators and whole-angle modes still has great potential to be explored in the future.

## Supplementary information

supplementary material

ALLAN

dynamic_drift.m

fourier_fit

fourier_fit2

model

scale_factor_nonlinearity_calculation

solve_a

data0035.mat

data01.mat

Data for Figure6a

Data for Figure6b_1

Data for Figure6b_2

Data for Figure6d

Figure6e_data1

Figure6e_data2

Figure6e_data3

figure6f_bias inatability1

figure6f_bias instability2

notes for running codes

Data for Figure2c

Data for Figure3c

Data for Figure3d

Data for Figure4b

Data for Figure6c

Data for Figure4c

Data for Figure4d,e

## References

[1] Li Q (2018). 0.04 degree-per-hour MEMS disk resonator gyroscope with high-quality factor (510 k) and long decaying time constant (74.9 s). Microsyst. Nanoeng..

[2] Giner J, Maeda D, Ono K, Shkel AM, Sekiguchi T (2018). MEMS gyroscope with concentrated springs suspensions demonstrating single digit frequency split and temperature robustness. J. Microelectromechanical Syst..

[3] Zaman MF, Sharma A, Hao Z, Ayazi F (2008). A mode-matched silicon-yaw tuning-fork gyroscope with subdegree-per-hour Allan deviation bias instability. J. Microelectromechanical Syst..

[4] Cho, J. Y. et al. 0.00016 deg/√ hr Angle Random Walk (ARW) and 0.0014 deg/hr Bias Instability (BI) from a 5.2 MQ and 1-cm Precision Shell Integrating (PSI) Gyroscope. In *2020 IEEE International Symposium on Inertial Sensors and Systems (INERTIAL)*. 1–4 (2020).

[5] Acar C, Shkel AM (2005). An approach for increasing drive-mode bandwidth of MEMS vibratory gyroscopes. J. Microelectromechanical Syst..

[6] Ahn CH (2014). Mode-matching of wineglass mode disk resonator gyroscope in (100) single crystal silicon. J. Microelectromechanical Syst..

[7] Sonmezoglu S, Alper SE, Akin T (2014). An automatically mode-matched MEMS gyroscope with wide and tunable bandwidth. J. Microelectromechanical Syst..

[8] Taheri-Tehrani P, Challoner AD, Horsley DA (2018). Micromechanical rate integrating gyroscope with angle-dependent bias compensation using a self-precession method. IEEE Sens. J..

[9] Woo, J. K. et al. Whole-angle-mode micromachined fused-silica birdbath resonator gyroscope (WA-BRG). In *2014 IEEE 27th International Conference on Micro Electro Mechanical Systems (MEMS)*. 20–23 (2014).

[10] Prikhodko IP, Zotov SA, Trusov AA, Shkel AM (2012). Foucault pendulum on a chip: rate integrating silicon MEMS gyroscope. Sens. Actuators A Phys..

[11] Lynch, D. D. MRIG frequency mismatch and quadrature control. In *2014 International Symposium on Inertial Sensors and Systems (ISISS)*. 1–4 (2014).

[12] Prikhodko, I. P. et al. Overcoming limitations of rate integrating gyroscopes by virtual rotation. In *2016 IEEE International Symposium on Inertial Sensors and Systems (INERTIAL)*. 5–8 (2016).

[13] Senkal D, Ahamed MJ, Trusov AA, Shkel AM (2013). Achieving sub-Hz frequency symmetry in micro-glassblown wineglass resonators. J. Microelectromechanical Syst..

[14] Nagourney, T. et al. 259 second ring-down time and 4.45 million quality factor in 5.5 kHz fused silica birdbath shell resonator. In *2017 19th International Conference on Solid-State Sensors, Actuators and Microsystems (TRANSDUCERS)*. 790–793 (2017).

[15] Shi Y (2020). Geometric imperfection characterization and precise assembly of micro shell resonators. J. Microelectromechanical Syst..

[16] Xiao D (2017). Fused silica micro shell resonator with T-shape masses for gyroscopic application. J. Microelectromechanical Syst..

[17] Lu, K. et al. Effective mechanical trimming of micro shell resonator with T-shape masses. In *2017 19th International Conference on Solid-State Sensors, Actuators and Microsystems (TRANSDUCERS)*. 1132–1135 (2017).

[18] Wang, Y. et al. Frequency split reduction by directional lapping of fused quartz micro wineglass resonators. In *2017 IEEE International Symposium on Inertial Sensors and Systems (INERTIAL)*. 78–81 (2017).

[19] Gregory, J. A. et al. Novel mismatch compensation methods for rate-integrating gyroscopes. In *Proceedings of the 2012 IEEE/ION Position, Location and Navigation Symposium*. 252–258 (2012).

[20] Hu Z, Gallacher B (2016). Extended Kalman filtering based parameter estimation and drift compensation for a MEMS rate integrating gyroscope. Sens. Actuators A Phys..

[21] Hu, Z. et al. Control and damping imperfection compensation for a rate integrating MEMS gyroscope. In *2015 DGON Inertial Sensors and Systems Symposium (ISS)*. 1–15 (2015).

[22] Lynch, D. D. Vibratory gyro analysis by the method of averaging. In *Proceedings 2nd St. Petersburg Conference on Gyroscopic Technology and Navigation, St. Petersburg*. 26–34 (1995).

[23] Kaajakari V, Mattila T, Oja A, Seppa H (2004). Nonlinear limits for single-crystal silicon microresonators. J. Microelectromechanical Syst..

[24] Agarwal M (2006). Optimal drive condition for nonlinearity reduction in electrostatic microresonators. Appl. Phys. Lett..

[25] Bowles SR, Gallacher BJ, Hu ZX, Fell CP, Townsend K (2014). Control scheme to reduce the effect of structural imperfections in a rate integrating MEMS gyroscope. IEEE Sens. J..

[26] Hu Z, Gallacher BJ (2018). Precision mode tuning towards a low angle drift MEMS rate integrating gyroscope. Mechatronics.

[27] Hu Z, Gallacher BJ (2019). Effects of nonlinearity on the angular drift error of an electrostatic MEMS rate integrating gyroscope. IEEE Sens. J..

[28] Li Q (2019). Nonlinearity reduction in disk resonator gyroscopes based on the vibration amplification effect. IEEE Trans. Ind. Electron..

[29] Vatanparvar, D. et al. Identification of Gain Mismatches in Control Electronics of Rate Integrating CVGs. In *2021 IEEE International Symposium on Inertial Sensors and Systems (INERTIAL)*. 1–4 (2021).

[30] Shi Y, Xi X, Li B, Chen Y, Lu K (2021). Micro hemispherical resonator gyroscope with teeth-like tines. IEEE Sens. J..

