# Supplementary material for: 0.79 ppm scale-factor nonlinearity whole-angle microshell gyroscope realized by real-time calibration of capacitive displacement detection
Source: Microsyst Nanoeng. 2021 Oct 13;7:79. doi: 10.1038/s41378-021-00306-6 (PMC8514555; doi:10.1038/s41378-021-00306-6)
Supplement: Supplementary file 1 — supplementary material [file 41378_2021_306_MOESM1_ESM.docx]

**Supplementary material**

**0.79 ppm scale factor nonlinearity whole-angle micro-shell gyroscope realized by real-time calibration of capacitive displacement detection**

Jiangkun Sun, Sheng Yu, Yongmeng Zhang*, Xiang Xi, Kun Lu, Yan Shi,

Qingsong Li, Dingbang Xiao*, Xuezhong Wu

National University of Defense Technology, Changsha, 410073, China

# 1. Dynamic model of micro-shell resonator gyroscope


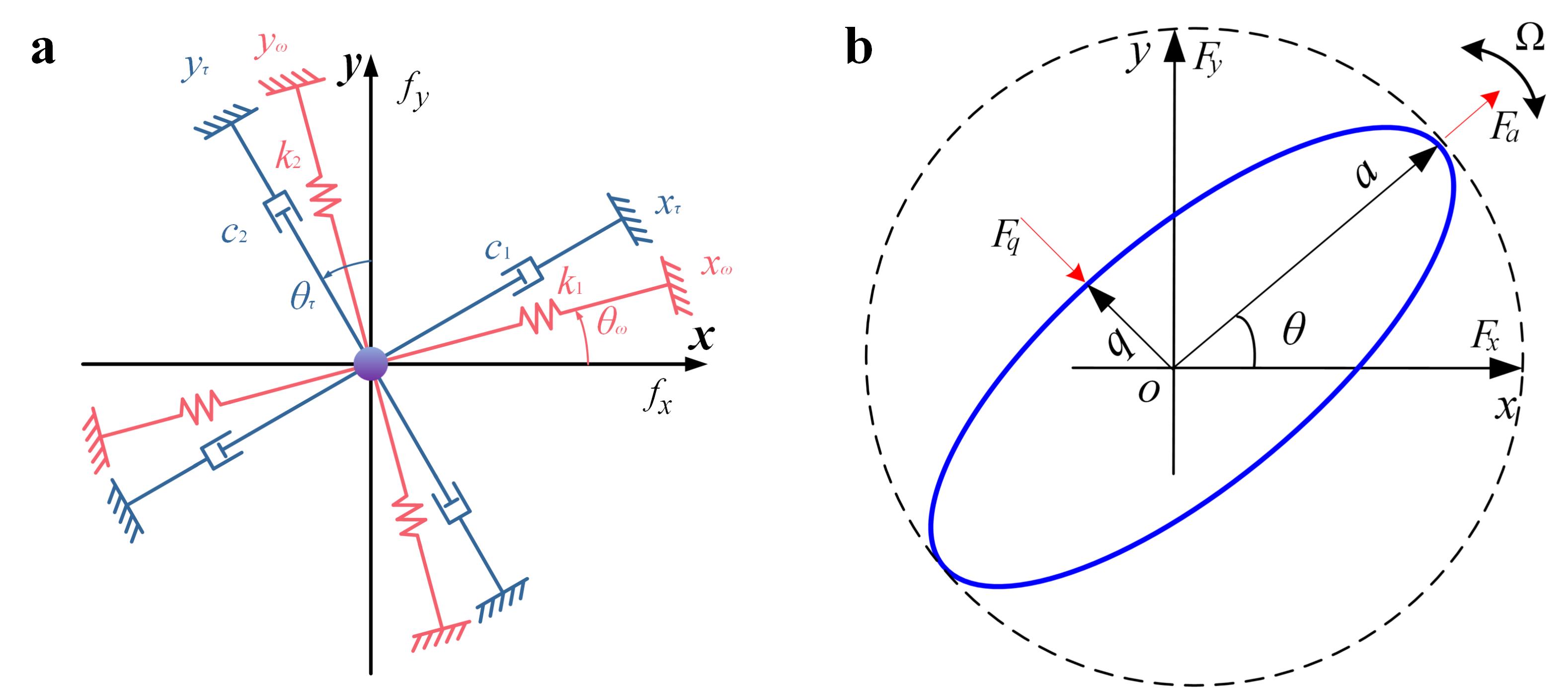


Fig. 1. (a) The two-dimensional harmonic oscillator (b) General elliptic orbit and procession of vibration modes.

For the two-dimensional spring-mass system shown in Fig.1(a), the equations can be expressed as

where$z=\left[\begin{matrix} x \\ y \end{matrix}\right],f=\left[\begin{matrix} f_{x} \\ f_{y} \end{matrix}\right],\sigma_{1}\text{=}\left[\begin{matrix} 0 & 1 \\ 1 & 0 \end{matrix}\right],\sigma_{2}=\left[\begin{matrix} 0 & 1 \\ -1 & 0 \end{matrix}\right],\sigma_{3}=\left[\begin{matrix} 1 & 0 \\ 0 & -1 \end{matrix}\right]$,$\frac{1}{\tau}=\frac{1}{2}\left(\frac{1}{\tau_{1}}+\frac{1}{\tau_{2}}\right),\Delta\left(\frac{1}{\tau}\right)=\frac{1}{\tau_{1}}-\frac{1}{\tau_{2}},$ $\omega^{2}=\frac{\omega_{1}^{2}+\omega_{2}^{2}}{2},\omega\Delta\omega=\frac{\omega_{1}^{2}-\omega_{2}^{2}}{2}$. *τ*_1_, *τ*_2_, *ω*_1_ and *ω*_2_ are decay times and resonant frequencies of two *n*=2 modes, *θ_τ_* and *θ_ω_* are the angles of the primary axes of damping and stiffness respectively. Ω is the input rotation rate and *κ* is the angle gain between the rotation of the gyroscope frame and vibration pattern, *f* is the input force.

The solution of equationis plotted in Fig.1(b), illustrating an elliptical orbit with major axis *a*, minor axis *q*, pattern angle *θ* and vibrating phase. Under the input rotation rate of Ω, the pattern angle tends to precess as

$$\theta_{1}=\theta_{0}+\int\left(-\kappa\Omega\right)dt$$

Therefore, the rotation angle Θ of the structure can be integrated and obtained directly from the variation of the pattern angle, which can be expressed as

$$\Theta=\int\Omega dt=-\frac{\theta_{1}-\theta_{0}}{\kappa}$$

The vibrations can also be expressed as

where *c_x_*, *c_y_*, *s_x_* and *s_y_* represent the in-phase and quadrature components of *x* and *y* respectively.

According to Lynch’s theory^1^, the control variables are

where *E* represents energy which is kept constant by the energy-control loop, *Q* represents quadrature which is nulled by quadrature-control loop, represents the phase difference between detection and actuation which is used in the phase-locked loop (PLL).

The pattern angle *θ* can be calculated from

$$\theta=\frac{1}{2}\arctan\left(\frac{S}{R}\right)$$

The equations of these control variables can be obtained by solving Eq.1. Using the method of averaging presented in the reference^1^, the equations can be described as

where

whererepresents the output of PLL, *F_a_* and *F_q_* represent the effective force at the major and minor axes of the ellipse, *f_ac_*, *f_as_*, *f_qc_* and *f_qs_* represent the in-phase and quadrature components of *F_a_* and *F_q_* respectively.

From the third equation in equation, we can know that stiffness asymmetry has no influence on the angle output of gyroscopes when the quadrature *Q* is suppressed to zero. Therefore, the angle-dependent angle drift is mainly from damping asymmetry, which is a 2*θ* harmonic component of the angle drift rate.

# 2. The effect of nonlinearity in detection

## 2.1 Nonlinear detection signal

The vibration signals are obtained by capacitance detection with carrier modulation, as shown in Fig. 2, where the frequency of carrier is much larger than the resonant frequency of gyroscopes.


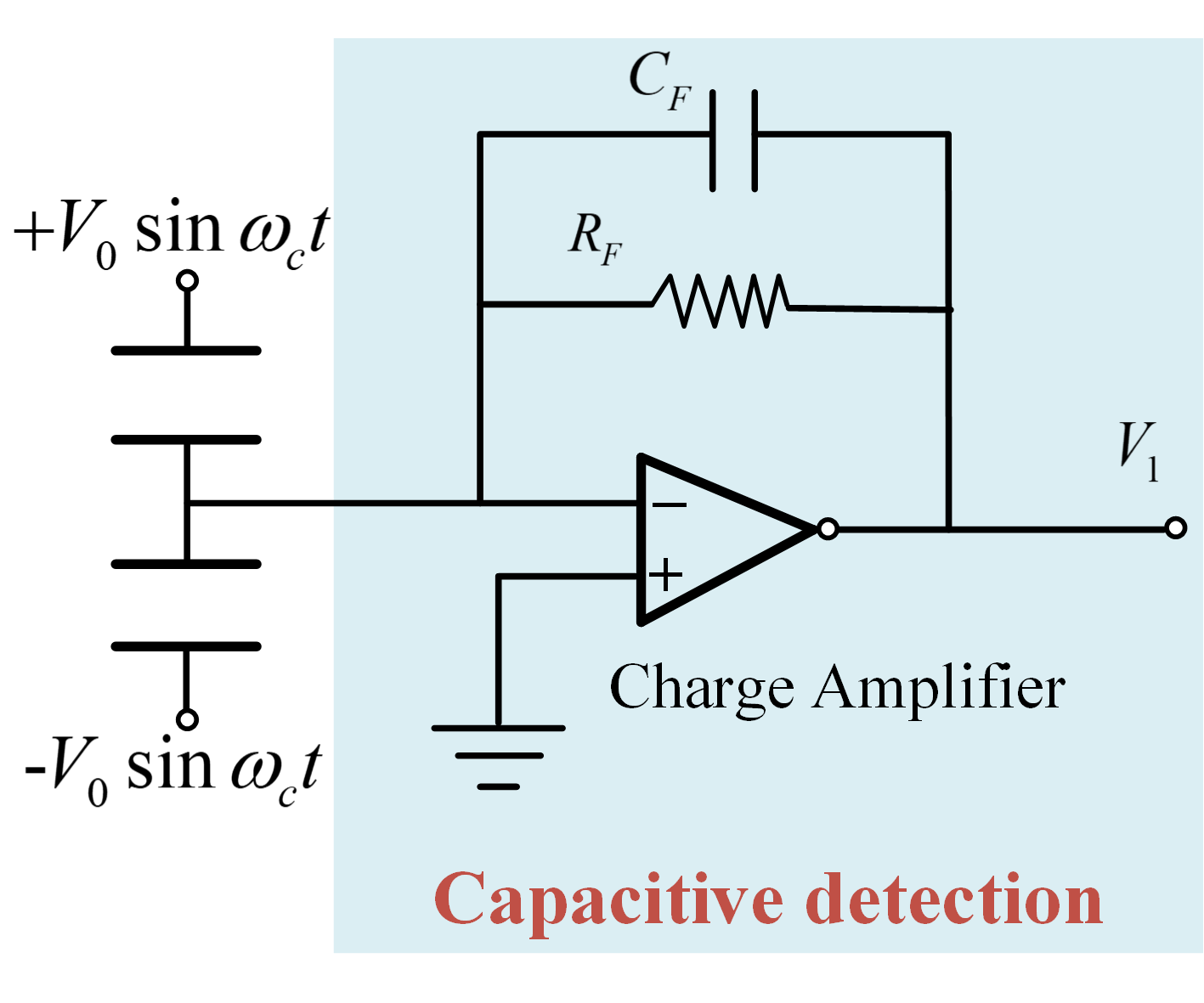


Fig. 2. Capacitance detection with carrier modulation

According to Fourier expansion, the differential capacitance is given by

$$\Delta C=\frac{\varepsilon A}{d_{0}-x}-\frac{\varepsilon A}{d_{0}+x}=\frac{2\varepsilon Ax}{d_{0}^{2}}\frac{1}{1-\left(\frac{x}{d_{0}}\right)^{2}}=\frac{2\varepsilon Ax}{d_{0}^{2}}\left[1+\left(\frac{x}{d_{0}}\right)^{2}+O\left(\frac{x}{d_{0}}\right)^{4}\right]\approx\frac{2\varepsilon A}{d_{0}^{2}}\left[1+\left(\frac{x}{d_{0}}\right)^{2}\right]x$$

where *ε* is the air dielectric constant, *d_0_* and *A* are the gap and area of electrodes and *x* represents the vibration displacement.

Therefore, the detection signal after carrier demodulation can be expressed as

$$V_{1}=-\frac{V_{0}}{C_{FB}}\Delta C=k_{s}\left[1+\left(\frac{x}{d_{0}}\right)^{2}\right]x$$

where$k_{s}=-\frac{2\varepsilon AV_{0}}{C_{FB}d_{0}^{2}}$.*V*_0_ represents the amplitude of the carrier and *C_FB_* represents the feedback capacitance.

## 2.2 Nonlinear gain in the demodulated signal

Supposing a vibration displacement

and substituting it into equation, one can obtain

After demodulation, other harmonic components will be removed except the first harmonic component. Hence, the demodulated signal is

where $G=k_{s}\left[1+\frac{3}{4}\left(\frac{x_{0}}{d_{0}}\right)^{2}\right]$. *G* represents the nonlinear gain in the demodulated signal.

Combining equation and , the demodulated in-phase and quadrature components of *x* and *y* can be expressed as

$$\begin{matrix} c_{x}=G_{x}a\cos\theta,s_{x}=-G_{x}q\sin\theta\\ c_{y}=G_{y}a\sin\theta,s_{y}=G_{y}q\cos\theta\end{matrix}$$

Where

$$\begin{matrix} G_{x}=k_{sx}\left[1+\frac{3}{4}\left(\frac{A_{x}}{d_{0x}}\right)^{2}\right],G_{y}=k_{sy}\left[1+\frac{3}{4}\left(\frac{A_{y}}{d_{0y}}\right)^{2}\right] \\ A_{x}^{2}=a^{2}\cos^{2}\theta+q^{2}\sin^{2}\theta,A_{y}^{2}=a^{2}\sin^{2}\theta+q^{2}\cos^{2}\theta\\ k_{sx}=-\frac{2\varepsilon A_{x}V_{0}}{C_{FB}d_{0x}^{2}},k_{sy}=-\frac{2\varepsilon A_{y}V_{0}}{C_{FB}d_{0y}^{2}} \end{matrix}$$

*k_sx_* and *k_sy_* represent the linear detection gain of two modes while *G_x_* and *G_y_* represent the nonlinear ones. *d_0x_* and *d_0y_* are the gaps of electrodes on the axes *x* and *y* respectively.

## 2.3 The effects of nonlinear detection gain

Combining equation and , the estimated control variables can be expressed as

From the second equation in equation, we can know that the minor axis *q* of elliptical orbit will be suppressed to zero by a quadrature-control loop despite the nonlinear detection gain. Therefore, equation can be simplified as

where$G_{x}=k_{sx}\left[1+3a^{2}\cos^{2}\theta/4d_{0x}^{2}\right],G_{y}=k_{sy}\left[1+3a^{2}\sin^{2}\theta/4d_{0y}^{2}\right]$.

From the last equation in equation , we can know that the PLL can still track the vibration phase of the gyroscope despite the nonlinear detection gain by holdingat zero. However, the control variables *E*, *R* and *S* will have angle-dependent calculating errors due to the nonlinear detection gain, leading to the angle-dependent major axis *a* of elliptical orbit and angle-estimated error *δθ* of pattern angle *θ*, which can be expressed as

$$\begin{matrix} a=\Lambda\left(\theta;E_{set}\right) \\ \delta\theta=\hat{\theta}-\theta=\frac{1}{2}\arctan\left(\frac{\hat{S}\left(\theta\right)}{\hat{R}\left(\theta\right)}\right)-\theta=\delta\theta\left(\theta\right) \end{matrix}$$

where *E_set_* is the desired value of the energy-control loop and$\Lambda\left(\theta;E_{set}\right)$is the solution of the following polynomial function in equation .

$$\begin{matrix} \left[\frac{9}{16}\left(\frac{k_{sx}^{2}}{d_{0x}^{2}}+\frac{k_{sy}^{2}}{d_{0y}^{2}}\right)\text{+}\frac{3}{4}\left(\frac{k_{sx}^{2}}{d_{0x}^{2}}-\frac{k_{sy}^{2}}{d_{0y}^{2}}\right)\cos2\theta+\frac{3}{16}\left(\frac{k_{sx}^{2}}{d_{0x}^{2}}+\frac{k_{sy}^{2}}{d_{0y}^{2}}\right)\cos4\theta\right]a^{4} \\ +\left[\frac{\left(k_{sx}^{2}+k_{sy}^{2}\right)}{2}+\frac{\left(k_{sx}^{2}-k_{sy}^{2}\right)}{2}\cos2\theta\right]a^{2}-E_{set}=0 \end{matrix}$$

From the equation above, we can not figure out the clear relationship of angle-estimated error *δθ* and pattern angle *θ*. Therefore, numerical analysis is carried out to illustrate the relationship. By normalization, let *E*_set_ equals to 0.01, namely *x*_0_*/d_0_*=0.1. The *δθ* can be calculated from the equation as shown in the Fig.3. Afterwards, the result is fitted by the equation $\delta\theta\text{=}asin(2(\theta+b))+csin(2(\theta+d))+e$. It is observed from the fitting results that the *δθ* is composed of 4*θ* harmonic component.


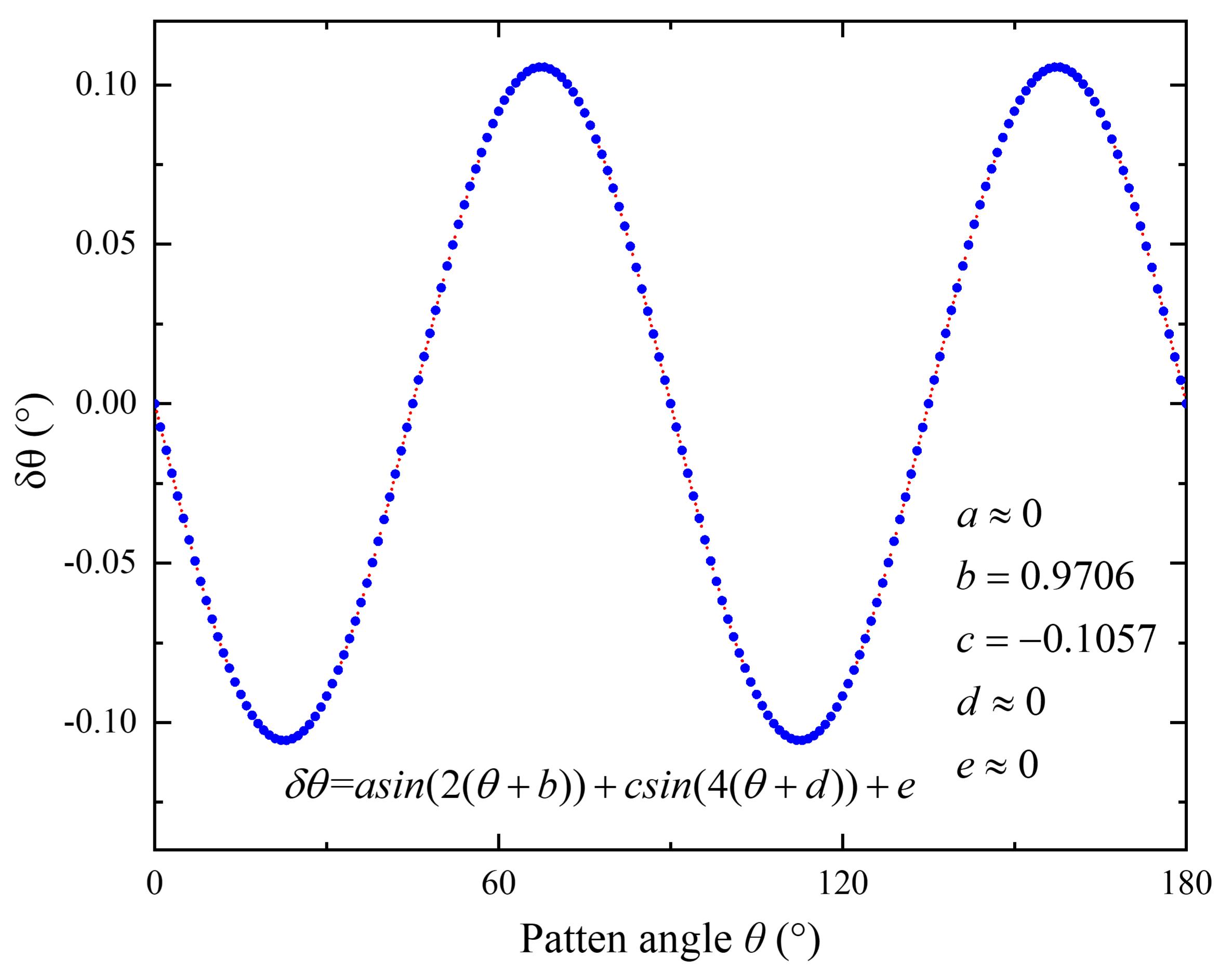


Fig. 3. Simulation results of *δθ* by numerical analysis

## 2.4 Angle drift due to nonlinearity in detection

Without the rate input, the angle drift due to nonlinearity in detection is mainly from a coupling of the energy-control loop and angle-control loop. Considering the angle-estimated error *δθ* of pattern angle *θ*, the equation can be modified as

$$\left[\begin{matrix} F'_{a} \\ F'_{q} \end{matrix}\right]=\left[\begin{matrix} \cos\delta\theta& -\sin\delta\theta\\ \sin\delta\theta& \cos\delta\theta\end{matrix}\right]\left[\begin{matrix} F_{a} \\ F_{q} \end{matrix}\right]$$

where$f'_{as}=\cos\delta\theta f_{as},f'_{qs}=\sin\delta\theta f_{as}$.

Substituting equation into equation , we can obtain

With the rate input Ω, equation should be modified as

where.

The rate output of the gyroscope can be expressed as

where.

Combining equation and , the angle drift rate due to capacitive displacement detection nonlinearity can be expressed as

# 3.Damping asymmetry compensation under whole-angle operation

As the analysis in the supplementary material, the damping asymmetry of the micro-shell resonator will result in the 2*θ* harmonic component of angle drift rate. It is observed from the equation below that the angle drift rate consists of two parts. The former one induced by damping asymmetry will only generate a 2*θ* harmonic component. However, the latter one coming from detection nonlinearity will produce a 4*θ* harmonic component due to the existence of $\delta\theta$.

Before compensating damping asymmetry, the angle drift rate should be measured at first under Ω=0. Afterwards, the result is fitted by the equation *y=a*sin*(*2*(x+b*))*+c*sin*(*4**(*x+d*))*+e* as shown in the Figure 3(a). To be specific, *a*sin*(*2**(*x+b*)) *and c*sin*(*4**(*x+d*)) representing 2*θ* and 4*θ* harmonic component of angle drift correspond to and respectively .

| 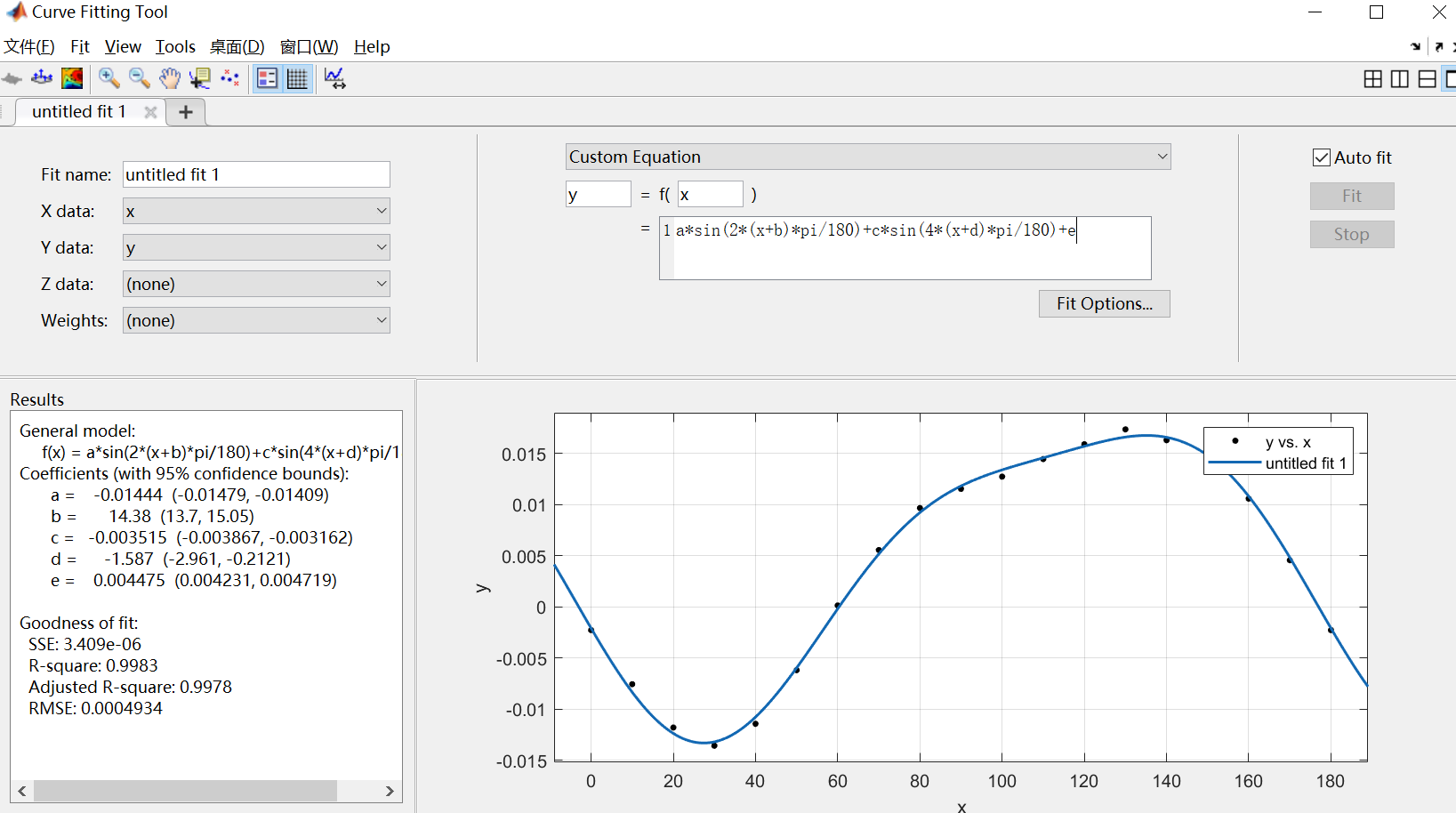 | 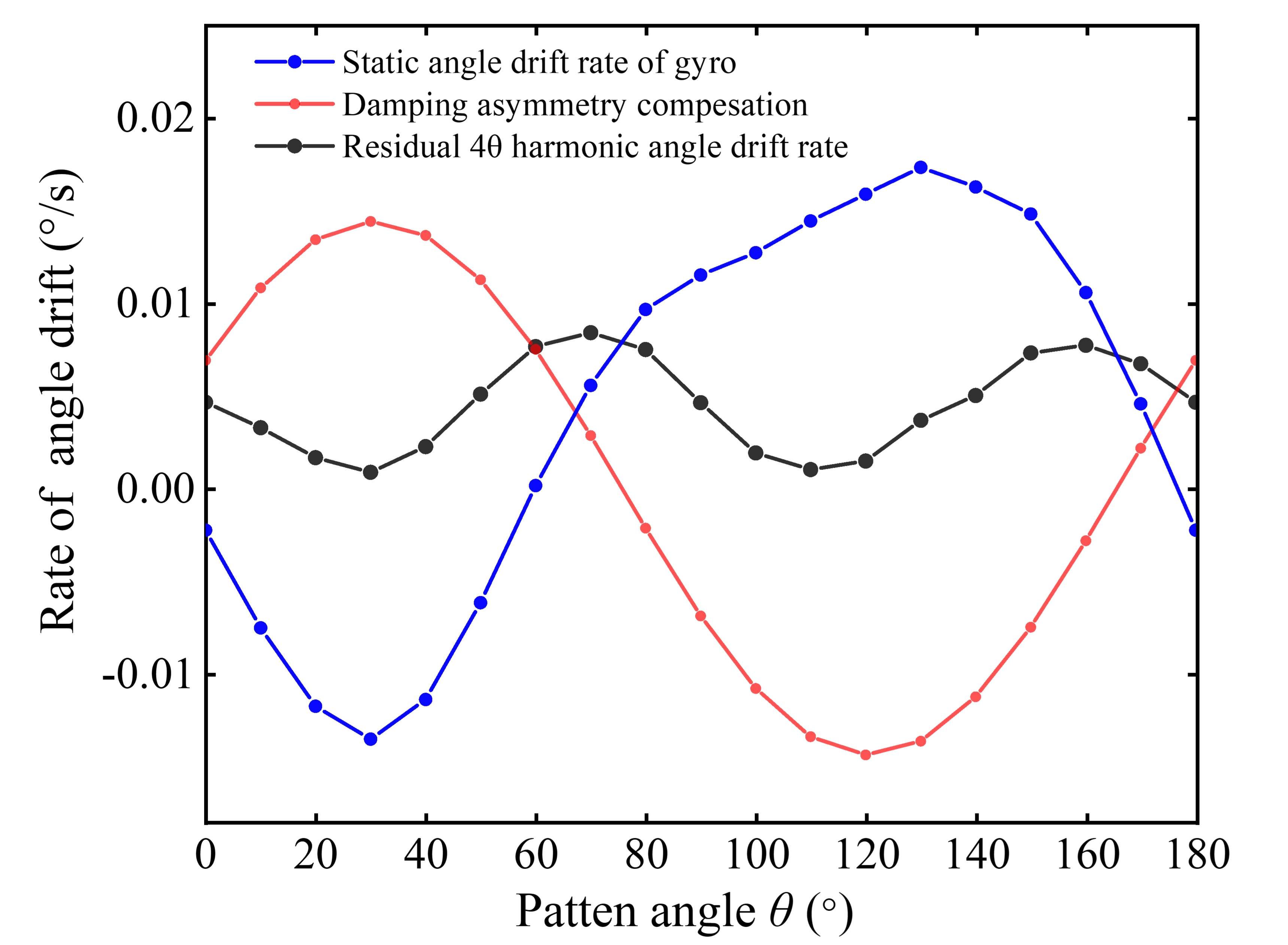 |
| --- | --- |
| Figure 3. (a) Curve fitting of static angle drift rate | (b) Results of damping asymmetry compensation |

According to the averaged equation in supplementary material, quadrature *Q* is suppressed to zero and the angle drift rate can be expressed as

From the equation above, we can compensate for the damping asymmetry by letting control force $f_{qs}\text{=}\omega\sqrt{E}\Delta\left(\frac{1}{\tau}\right)\sin2\left(\theta-\theta_{\tau}\right)$. The fitting results above provide that $\Delta\left(\frac{1}{\tau}\right)\text{=}\frac{2a\pi}{180}$and $\theta_{\tau}\text{=-b}$. The 2*θ* harmonic component of angle drift rate can be removed by damping asymmetry compensation and the residual 4*θ* harmonic component $\frac{1}{\tau}\tan\delta\theta$ can be observed as shown in Figure 4(b).

1 Lynch, D. D. in *Proc. 2nd St. Petersburg Conf. on Gyroscopic Technology and Navigation, St. Petersburg.* 26-34.
